# DMRT1 repression using a novel approach to genetic manipulation induces testicular dysgenesis in human fetal gonads

**DOI:** 10.1093/humrep/dey289

**Published:** 2018-09-29

**Authors:** Joni Macdonald, Karen R Kilcoyne, Richard M Sharpe, Áine Kavanagh, Richard A Anderson, Pamela Brown, Lee B Smith, Anne Jørgensen, Rod T Mitchell

**Affiliations:** 1 MRC Centre for Reproductive Health, The Queen’s Medical Research Institute, The University of Edinburgh, 47 Little France Crescent, Edinburgh, Scotland, UK; 2 School of Environmental and Life Sciences, Faculty of Science, University of Newcastle, Callaghan, NSW, Australia; 3 University Department of Growth and Reproduction, Rigshospitalet, Blegdamsvej 9, Copenhagen, Denmark; 4 Edinburgh Royal Hospital for Sick Children, 9 Sciennes Road, Edinburgh, Scotland, UK

**Keywords:** human fetal testis, gonadal development, DMRT1, testicular dysgenesis, sex differentiation, miRNA

## Abstract

**STUDY QUESTION:**

Does loss of DMRT1 in human fetal testis alter testicular development and result in testicular dysgenesis?

**SUMMARY ANSWER:**

DMRT1 repression in human fetal testis alters the expression of key testicular and ovarian determining genes, and leads to focal testicular dysgenesis.

**WHAT IS KNOWN ALREADY:**

Testicular dysgenesis syndrome (TDS) is associated with common testicular disorders in young men, but its etiology is unknown. DMRT1 has been shown to play a role in the regulation of sex differentiation in the vertebrate gonad. Downregulation of DMRT1 in male mice results in trans-differentiation of Sertoli cells into granulosa (FOXL2^+^) cells resulting in an ovarian gonadal phenotype.

**STUDY DESIGN, SIZE, DURATION:**

To determine the effect of DMRT1 repression on human fetal testes, we developed a novel system for genetic manipulation, which utilizes a Lentivral delivered miRNA during short-term *in vitro* culture (2 weeks). A long-term (4–6 weeks) *ex vivo* xenograft model was used to determine the subsequent effects of DMRT1 repression on testicular development and maintenance. We included first and second-trimester testis tissue (8–20 weeks gestation; *n* = 12) in the study.

**PARTICIPANTS/MATERIALS, SETTING, METHODS:**

Human fetal testes were cultured in vitro and exposed to either of two DMRT1 miRNAs (miR536, miR641), or to scrambled control miRNA, for 24 h. This was followed by a further 14 days of culture (*n* = 3–4), or xenografting (*n* = 5) into immunocompromised mice for 4–6 weeks. Tissues were analyzed by histology, immunohistochemistry, immunofluorescence and quantitative RT-PCR. Endpoints included histological evaluation of seminiferous cord integrity, mRNA expression of testicular, ovarian and germ cell genes, and assessment of cell number and protein expression for proliferation, apoptosis and pluripotency factors. Statistical analysis was performed using a linear mixed effect model.

**MAIN RESULTS AND THE ROLE OF CHANCE:**

DMRT1 repression (miR536/miR641) resulted in a loss of DMRT1 protein expression in a sub-population of Sertoli cells of first trimester (8–11 weeks gestation) human fetal testis; however, this did not affect the completion of seminiferous cord formation or morphological appearance. In second-trimester testis (12–20 weeks gestation), DMRT1 repression (miR536/miR641) resulted in disruption of seminiferous cords with absence of DMRT1 protein expression in Sertoli (SOX9^+^) cells. No differences in proliferation (Ki67^+^) were observed and apoptotic cells (CC3^+^) were rare. Expression of the Sertoli cell associated gene, *SOX8*, was significantly reduced (miR536, 34% reduction, *P* = 0.031; miR641 36% reduction, *P* = 0.026), whilst *SOX9* expression was unaffected. Changes in expression of *AMH* (miR536, 100% increase, *P* = 0.033), *CYP26B1* (miR641, 38% reduction, *P* = 0.05) and *PTGDS* (miR642, 30% reduction, *P* = 0.0076) were also observed. Amongst granulosa cell associated genes, there was a significant downregulation in *R-spondin 1* expression (miR536, 76% reduction, *P* < 0.0001; miR641, 49% reduction, *P* = 0.046); however, there were no changes in expression of the granulosa cell marker, *FOXL2*. Analysis of germ cell associated genes demonstrated a significant increase in the expression of the pluripotency gene *OCT4* (miR536, 233%, *P* < 0.001). We used the xenograft system to investigate the longer-term effects of seminiferous cord disruption via DMRT1 repression. As was evident *in vitro* for second-trimester samples, DMRT1 repression resulted in focal testicular dysgenesis similar to that described in adults with TDS. These dysgenetic areas were devoid of germ cells, whilst expression of FOXL2 within the dysgenetic areas, indicated trans-differentiation from a male (Sertoli cell) to female (granulosa cell) phenotype.

**LIMITATIONS, REASONS FOR CAUTION:**

Human fetal testis tissue is a limited resource; however, we were able to demonstrate significant effects of DMRT1 repression on the expression of germ and somatic cell genes, in addition to the induction of focal testicular dysgenesis, using these limited samples. *In vitro* culture may not reflect all aspects of human fetal testis development and function; however, the concurrent use of the xenograft model which represents a more physiological system supports the validity of the *in vitro* findings.

**WIDER IMPLICATIONS OF THE FINDINGS:**

Our findings have important implications for understanding the role of DMRT1 in human testis development and in the origin of testicular dysgenesis. In addition, we provide validation of a novel system that can be used to determine the effects of repression of genes that have been implicated in gonadal development and associated human reproductive disorders.

**STUDY FUNDING/COMPETING INTEREST(S):**

This project was funded by a Wellcome Trust Intermediate Clinical Fellowship (Grant No. 098522) awarded to RTM. LBS was supported by MRC Programme Grant MR/N002970/1. RAA was supported by MRC Programme Grant G1100357/1. RMS was supported by MRC Programme Grant G33253. This work was undertaken in the MRC Centre for Reproductive Health which is funded by the MRC Centre grant MR/N022556/1. The funding bodies had no input into the conduct of the research or the production of this manuscript. The authors have declared no conflicts of interest.

## Introduction

Development of the mammalian testis and ovary from the bi-potential gonad occurs through sex-specific differentiation of somatic cell populations which support the development of the gonad into a testis or an ovary. Key to this process is the differentiation of a population of somatic precursor cells into either Sertoli cells (testis) or pre-granulosa cells (ovary). In males, this process is primarily directed by the presence of a Y-chromosome, and subsequent gonadal development is under the control of several signaling cascades working in concert to drive cell lineage specification in the testis (Stevant *et al.*, 2018): In females, specific cascades are also involved in determining ovarian development (Pannetier *et al.*, 2016). Once testicular somatic cell populations are established, testosterone production from fetal Leydig cells is initiated to induce masculinization of the fetus. Failure of normal sex-determination or perturbed hormone production during fetal gonadal development in humans can result in disorders of sex development (DSD) (Jørgensen *et al.*, 2015a; Hersmus *et al.*, 2017) and is also implicated in the development of testicular dysgenesis syndrome (TDS; cryptorchidism, hypospadias, testicular cancer and low sperm counts; Skakkebaek *et al.*, 2016; van den Driesche *et al.*, 2017).

Our earlier understanding of gonadal somatic cell development was that, once specified, Sertoli and granulosa cell populations were fixed. However, it has recently been demonstrated in mice that loss of expression of key genes in postnatal life can re-programme Sertoli cells into granulosa cells and vice-versa, demonstrating a plasticity of gonadal fate long after normal formation of a testis or ovary (Uhlenhaut *et al.*, 2009; Matson *et al.*, 2011). *DMRT1* (Doublesex and mab-3 related transcription factor 1) is a conserved autosomal gene expressed by both the Sertoli and germ cells in the developing mammalian testis (Raymond *et al.*, 1998, 1999a). DMRT1 has been shown to play a role in the regulation of sex determination in the vertebrate gonad (Matson and Zarkower. 2012) and also during the mitosis to meiosis switch in germ cells (Matson *et al.*, 2010, Jorgensen *et al.*, 2012). In mice, DMRT1 expression in Sertoli cells represses ovarian determining genes such as *FOXL2* via the retinoic acid pathway (Minkina *et al.*, 2014). Conversely, downregulation of DMRT1 in males during prepuberty or adulthood results in upregulation of *FOXL2* and trans-differentiation of Sertoli cells into granulosa-like cells, resulting in an ovarian gonadal phenotype, including estrogen production (Matson *et al.*, 2010). Studies using *DMRT1* null mice showed that despite the lack of DMRT1 expression, mice are born phenotypically male and it is not until after birth that they undergo sex reversal (Raymond *et al.*, 2000; Matson *et al.*, 2011). These studies indicate that, in contrast to DMRT1 homologs in other vertebrates, mammalian DMRT1 is not involved in the initial sex determination, but is instead required for maintaining male gonadal fate (Matson and Zarkower. 2012). In humans, mutations and/or deletions in the short arm of Chromosome 9, where *DMRT1* is located, can result in varying degrees of sex reversal (Hoo *et al.*, 1989; Bennett *et al.*, 1993; Vinci *et al.*, 2007) and 46,XY individuals with *DMRT1* mutations may present with DSD or features of TDS (Murphy *et al.*, 2015).

Much of our understanding of mammalian gonadal development is derived from studies in mice. However, in humans, the genes and signaling networks that control specification of the fetal testis and whether there is plasticity in somatic cell differentiation are not well established. Research into the human relevance of findings from rodent studies has been hampered by the lack of dynamic experimental model systems that can determine the effect of genetic manipulation on subsequent development in the human fetal gonad.

In the present study, we describe a novel system for genetic manipulation in the human fetal testis, which utilizes short-term *in vitro* culture followed by long-term xenografting to determine the effects of gene repression on subsequent testicular development and maintenance. Using this system, we show that DMRT1 can be successfully repressed in Sertoli cells of first and second-trimester fetal testes with resulting effects on the expression of key testicular and ovarian determining genes, and leading to focal testicular dysgenesis in the human fetal testis.

## Materials and Methods

### Experimental design

Given the evidence from rodent studies for effects of DMRT1 repression on testicular development, we aimed to determine whether experimentally-induced repression of DMRT1 results in aberrant development of the human fetal testis. Effects of DMRT1 repression was studied in human fetal testes taken from different stages of pregnancy using hanging drop culture (first and second-trimester testes) and a xenograft system (second-trimester testes). Samples were exposed to scrambled miRNA (control) or either of two miRNAs that targeted different regions of the human DMRT1 gene. Endpoints evaluated included histological evaluation of seminiferous cord formation and maintenance, mRNA expression levels of testicular, ovarian and germ cell genes known to be important in gonad development and assessment of cell number, proliferation status and of pluripotency marker expression. Inclusion criteria and measured endpoints were defined before the start of the study. For human fetal gonad experiments, the sample sizes (*n* = 4–8) were based on those required to achieve statistical significance in previous studies using the same methodology (van den Driesche *et al.*, 2015). The study ended once the required number of experiments had been conducted, and data were analyzed after the cessation of the study. For fluorescence immunohistochemistry, all samples from the same experiment were stained in the same run. Cell counts were determined with the investigator blind to the treatment group. To compare the effects of treatment versus vehicle in xenografts for each individual human fetal testis, we grafted tissue from each fetus into replicate host mice (*n* = 3–6) and randomly allocated these mice to receive tissue that had been exposed to either scrambled miRNA, miR536 or miR641.

### Ethics statement and sample collection

Ethical approval for receipt and use of human fetal tissues was obtained from the South East Scotland Research Ethics Committee (LREC08/S1101/1) and NRES committee North East – Newcastle and North Tyneside 1 (08/H0906/21 + 5) and written and informed consent was provided by the pregnant women. First- and second-trimester human fetal testes (8.5–20 weeks gestation; *n* = 12) were obtained after medical termination of pregnancy as described previously (Mitchell *et al.*, 2008). None of the terminations were due to fetal abnormalities. Gestation was determined by ultrasound scan and subsequent direct measurement of foot length. The sex of first trimester testes was confirmed by PCR for the male-specific gene *SRY*. To permit subsequent gene expression analysis, where possible a small sample of gonad tissue was removed, snap frozen and stored at −70°C until RNA extraction. The remainder was placed immediately into medium containing Leibovitz L-15 with added glutamine, 10% fetal bovine serum, 1% penicillin/streptomycin and 1% non-essential amino acids (all Sigma-Aldrich).

For studies involving animals, specific approval, including ethical approval, was given by the UK Home Office and all procedures were carried out in accordance with the Animal (Scientific procedures) Act 1986.

### Viral transduction of HEK293T cells

Recombined vectors pLent6.2_cppt_ CMV_mCherry_miR-NEG_control containing sequences encoding scrambled miRNA (negative control), and pLent6.2_cppt_CMV_mCherry_rDMRT1_miR536 and miR641, two miRNA targeted against human *DMRT1*, were created and transfected into HEK293T cells to produce lentiviral particles Lv-cppt-CMV-mCherry-miR-NEG control (2.7 × 10^6^ TU/ml), Lv-cppt-CMV-mCherry-rDMRT1-miR536 (7 × 10^6^ TU/ml) and miR641 (5.1 × 10^6^ TU/ml). Briefly, DMRT1-miRNA oligonucleotides were designed using the Invitrogen BLOCK-iT™ RNAi designer web programme to identify DNA sequences within rat *DMRT1* (transcript variant 1 accession no. NM_000044), labeled according to their position from the translational start site. These and the non-targeting sequence for the Scrambled miRNA are shown in Supplementary Table S1. Targeting sequences were initially designed based on rat *DMRT1* with subsequent confirmation of similarity for the human *DMRT1* sequence. Rat *DMRT1* (rDMRT1) miRNA and Scrambled miRNA oligo constructs were each cloned into a pcDNA6.2_GW_EmGFP_miR Gateway plasmid (Invitrogen) using the BLOCK-iT™ Pol II miR RNAi Expression Vector Kit (Invitrogen, K4935-00) according to the manufacturer’s protocols, and the inserts were verified by DNA sequencing before recombining into pLent6.2 vectors. HEK293T cells were transfected with the pLent6.2 vectors to produce the Lentivirus particles using the BLOCK-iT™ Lentiviral Pol II miR RNAi Expression System (Invitrogen, K4937-00).

### Viral transduction of tissue in a ‘hanging drop’ culture system

Following fetal gonadal dissection, the testis was cut into small pieces (~1 mm^3^). Tissue pieces were placed in ‘hanging drop’ culture in medium spiked with the appropriate virus preparation in a total volume of 30μl/tissue piece, for 24 h at 37°C under 5% CO_2_ (Jørgensen *et al.*, 2015b). Tissue was then either used for xenografting or transferred to fresh culture medium containing alpha MEM (Lonza), 10% fetal bovine serum, 1% penicillin/streptomycin, 1% non-essential amino acids, 2 mM l-glutamine, 2 mM sodium pyruvate and 1% insulin transferrin selenium (ITS) (all Sigma-Aldrich). The latter tissue was then maintained in culture for 13 days, with medium changed every 2 days.

### Human tissue xenografting

#### Animals

Castrate CD1-nude (host) mice were used for xenografting studies. Castration was performed by scrotal incision under anesthesia (inhalation of isoflurane) at least two weeks prior to xenografting. For 3 days post-castration, the mice were administered an analgesic (Carprofen, Pfizer) in their drinking water.

#### Human tissue and xenografting procedure

As previously described (Mitchell *et al.*, 2010), second-trimester human fetal testes (*n* = 5) were grafted subcutaneously into host CD1-nude mice using a 13-gauge cancer implant needle (Popper and sons). For each fetus, 4–6 pieces (~1 mm^3^) of testis tissue was inserted under the dorsal skin of the host mouse, and grafts were distributed evenly along either side of the midline. A total of 3–6 mice were grafted per fetus, and the mice were maintained for 4–6 weeks post grafting before retrieval of the tissue.

#### Xenograft retrieval

Host mice were killed by cervical dislocation and the retrieved xenografts were fixed in Bouin’s fluid (Clin-Tech). Post fixation, samples were paraffin embedded, sectioned and assessed by H&E or used for immunohistochemistry as below.

### Immunohistochemistry

Immunohistochemistry was carried out using standard immunohistochemical and immunofluorescent techniques as previously described (Fisher *et al.*, 2003; Mitchell *et al.*, 2008). Primary antibodies are described in Supplementary Table SII. Images were captured either using an Olympus Provis AX70 light microscope or in the case of fluorescent images a Zeiss LSM 510 Axio Observer Z1 confocal laser microscope (Carl Zeiss Ltd.). All images were compiled using Zen (Blue edition, Carl Zeiss Ltd.), Photoshop 9.0 (Adobe Systems Inc.). The total number of proliferative (Ki67) or apoptotic (Cleaved Caspase 3) positive stained cells was quantified and expressed relative to the total tissue section area for all treatment groups (*n* = 4–6) in first trimester hanging drop cultures.

### Gene expression analysis

Total RNA was extracted from human fetal gonads using the RNeasy Micro Kit with on-column DNase digestion (Qiagen, UK), as per the manufacturer’s instructions. Concentration and purity of the extracted RNA was assessed using a Nanodrop 1000 spectrophotometer (Thermo Fisher Scientific inc). cDNA was prepared using the maxima first strand cDNA synthesis kit (Thermo Fisher Scientific inc). Gene expression using specific human primers (Supplementary Table SIII) was determined using SYBR Green PCR Master Mix (Qiagen) to carry out Quantitative real time PCR (qRT-PCR) on the ABI 7900HT (Applied Biosystems). ABI7900HT runs consisted of a hot start at 95°C (3 min) followed by 40 cycles of 95°C (5 sec) and 65°C (15 sec). Melt curve analysis was performed as described previously (Bayne *et al.*, 2004) to confirm specific PCR products. Standard curve analysis, with increasing dilutions (from 1:10 to 1:640) of control cDNA were used to confirm the efficiency of the PCR amplification. All slopes of the standard curves were close to −3.3 (equivalent to 100% PCR efficiency or two-fold amplification per cycle), allowing quantification using the 2^−ΔΔCt^ method (Bayne *et al.*, 2004). Expression levels were normalized to *RPS20* and *RPS29* (both validated for use with human testis tissues) and also to SOX9 (Sertoli cells) expressed as a ratio with control tissue set to 1 (Svingen *et al.*, 2014).

### Statistics

To take into account the inter-individual variation between fetuses, the results were analyzed using a linear mixed effects model in R (www.r-project.org). Testicular pieces were randomly allocated to receive either the scrambled miRNA virus or one of the DMRT1-miRNA viral constructs. Significance was set at *P* < 0.05 and no outliers were excluded.

## Results

### DMRT1 repression does not affect seminiferous cord formation in the first trimester human fetal testis

**Figure 1:**
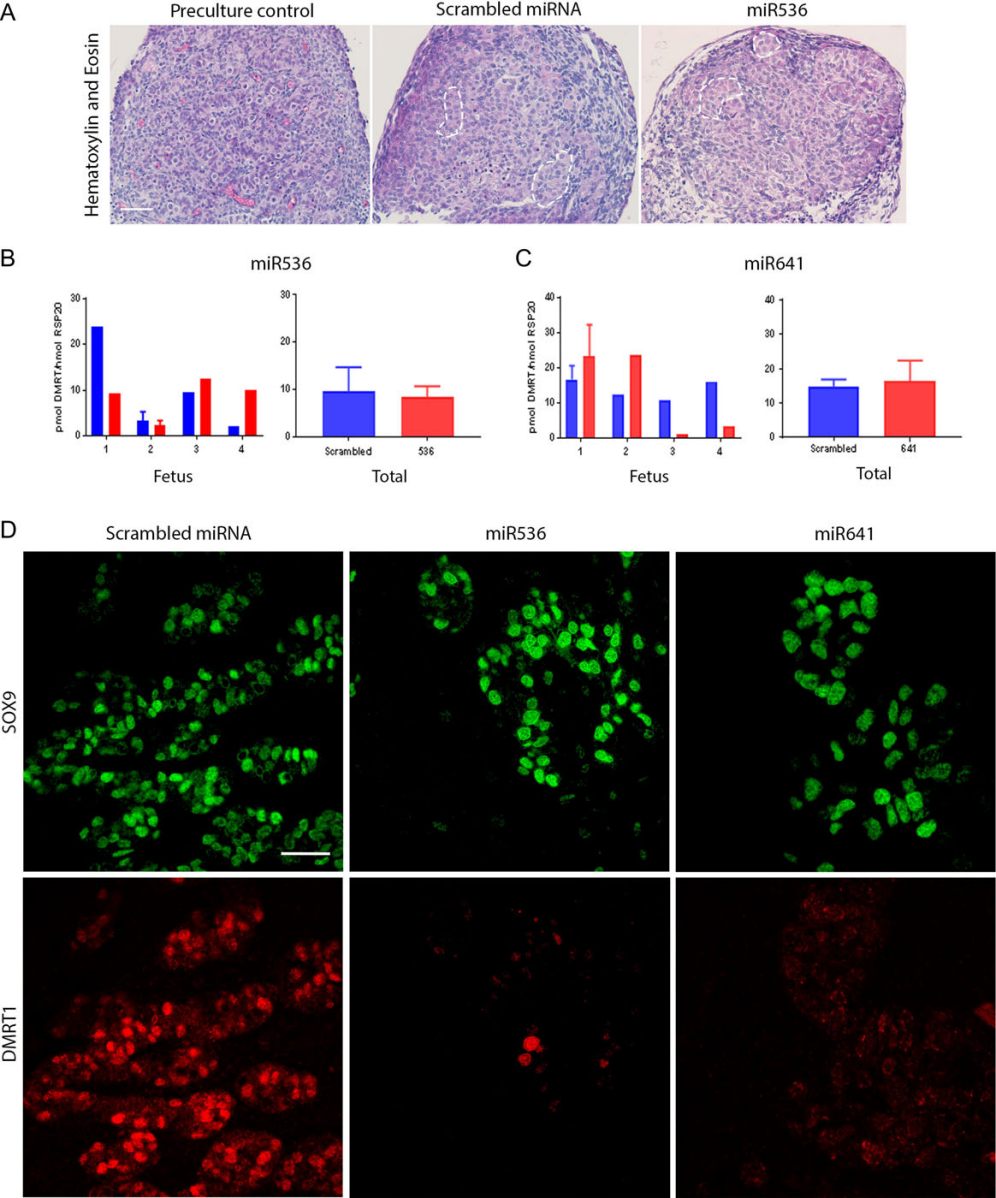
DMRT1 knockdown does not affect seminiferous cord formation in the first trimester human fetal testis.(**A**) H + E staining of pre-culture control (left panel) and after culture for 13 days following an initial 24 h lentiviral exposure to scrambled miRNA (middle panel) or miR536 (right panel). Seminiferous cords are delineated by broken white lines. *DMRT1* mRNA expression in (**B**) miR536- and (**C**) miR641-exposed tissues compared to scrambled miRNA controls (*n* = 4 for each DMRT1-miRNA exposure group). (**D**) Immunofluorescence analysis of SOX9 (green) and DMRT1 (red) expression in Sertoli cells in fetal testis tissue following transduction with either scrambled miRNA (left panel), miR536 (middle panel) or miR641 (right panel) lentiviral constructs. Scale bars: (A) 20 μM and (D) 50 μM. Graphs show mean ± SEM. Data analyzed using a linear mixed effects model in R.

First trimester human fetal testis tissue was exposed to lentiviral particles containing sequences that encoded either a scrambled miRNA (control) or one of two miRNAs targeted against *DMRT1* (miR536 and miR641). Using the hanging drop culture system, testis pieces were exposed to their respective miRNA for 24 h, followed by maintenance in standard culture medium for a further 13 days. Initial histological assessment of the tissue was carried out to assess survival and cellular development, which demonstrated normal gross morphology after culture (Fig. 1A). Cell counts for proliferating (Ki67^+^) and/or apoptotic (Cleaved Caspase 3^+^) cells showed no significant difference between the control and DMRT1-targeted miRNA samples (Supplementary Fig. S1A).

*DMRT1* gene expression in the first trimester fetal testis tissue, after culture, was quantified using RT-qPCR (Fig. 1B,C). Due to considerable inter-individual variation between fetuses, no significant difference in DMRT1 gene expression was observed in tissues exposed to either miR536 or miR641 when compared to scrambled miRNA-exposed control tissue. Given that miRNA-induced changes in gene expression are not always detectable at the transcript level, we used double immunofluorescence to determine DMRT1 protein expression. Sertoli cells (SOX9^+^) devoid of DMRT1 protein expression were frequently observed in both the miR536 and miR641-exposed tissue, indicating effective repression, whilst in comparison DMRT1 expression was present in all of the Sertoli cells of scrambled miRNA controls (Fig. 1D).

Seminiferous cords had not yet formed in the first trimester controls; however, cord formation could be clearly identified in all of the tissues exposed to the scrambled control and also in those in which partial repression of DMRT1 expression had been achieved, indicating that DMRT1 is not required for seminiferous cord formation (Fig. 1A).

### Repression of DMRT1 in second-trimester human fetal testis alters expression of genes involved in testicular and ovarian development

**Figure 2:**
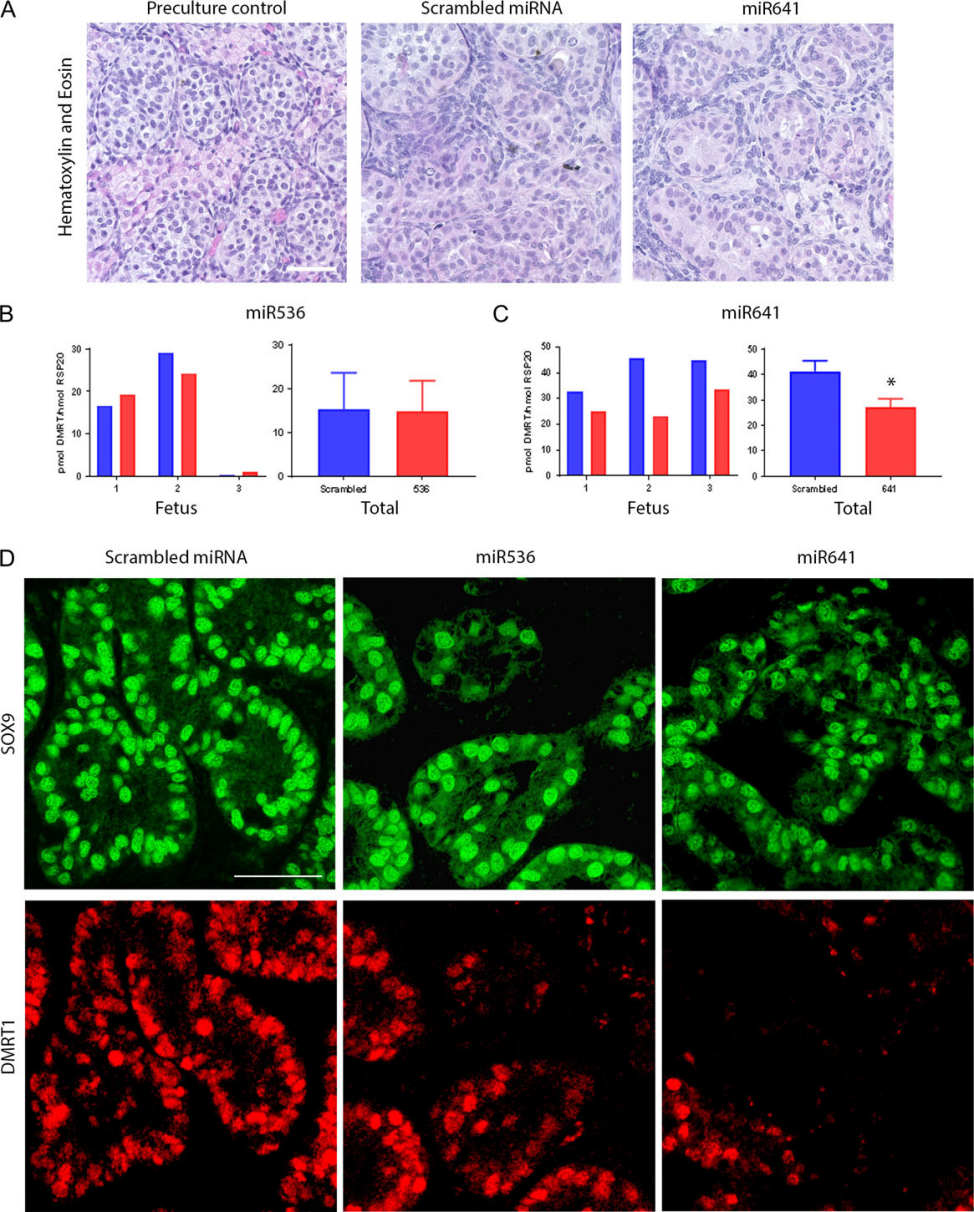
DMRT1 expression is lost in Sertoli cells of the second-trimester human fetal testis after in vitro exposure to miR536 and miR641. H + E staining of pre-culture control (left panel) and after culture for 13 days following an initial 24 h lentiviral exposure to scrambled miRNA (middle panel) or miR641 (right panel). Normal histological appearance of the testicular tissue is demonstrated. *DMRT1* mRNA expression in (**B**) miRNA536- and (**C**) miRNA641-exposed tissues compared to scrambled miRNA controls (*n* = 3 for each DMRT1-miRNA treatment group, *P < 0.05). (**D**) Immunofluorescence analysis of SOX9 (green) and DMRT1 (red) expression in Sertoli cells in fetal testis tissue following transduction with either scrambled miRNA (left panels), miR536 (middle panels) or miR641 (right panels) lentiviral constructs. Scale bar (A) 20 μM and (D) 50 μM. Graphs show mean ± SEM. Data analyzed using a linear mixed effects model in R.

To investigate the effects of DMRT1 repression in second-trimester fetal testis cultures, we used the same approach as described above for first trimester tissues. In control tissues, seminiferous cords had already formed and cord structure was largely maintained in the tissue over the culture period for both scrambled and DMRT1-miRNA (Fig. 2A). Similar to first trimester tissue, proliferation (Ki67^+^ cells) was observed in a large proportion of cells within the testis, with no discernible difference between DMRT1 repression or control tissue. Apoptotic (Cleaved Caspase 3^+^) cells were rarely identified (Supplementary Fig. S1B). These results are consistent with previously published work using non-exposed human fetal gonadal tissue under similar culture conditions (Jørgensen *et al.*, 2015b).

Analysis of the mRNA expression levels of DMRT1 in the tissue at the end of the culture period was quantified using RT-qPCR (Fig. 2B,C). Again, no significant difference in *DMRT1* mRNA was observed for miR536-exposed tissue compared with scrambled miRNA-exposed controls (Fig. 2B); however, there was a significant reduction in *DMRT1* gene expression (34%, *P* = 0.018) in tissue exposed to miR641 virus compared to scrambled controls (Fig. 2C). Similar to first trimester tissue, there was clear inter-individual variation between fetuses. Using fluorescence immunohistochemistry, we observed a loss of DMRT1 protein expression in Sertoli (SOX9^+^) cells in testes exposed to either miR536 or miR641 (Fig. 2D).

**Figure 3:**
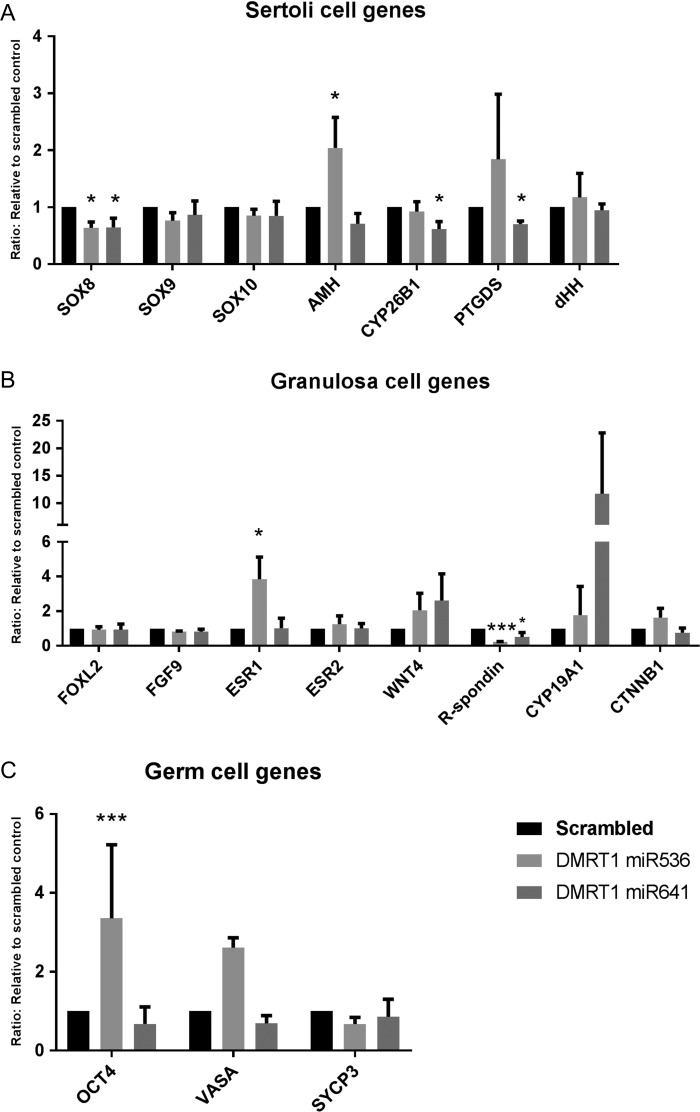
DMRT1 knockdown in second-trimester human fetal testis alters expression of genes involved in testicular and ovarian development. RT-qPCR analysis of genes associated with development of (**A**) Sertoli (**B**) granulosa and (**C**) germ cells in second-trimester human fetal testes tissue culture for 13 days after an initial 24 h exposure to scrambled miRNA, miR536 or miR641. Expression was normalized to RPS29 (*n* = 2–3 per treatment group, *P < 0.05, **P < 0.01, ***P < 0.001). Graphs show mean ± SEM. Data analyzed using a linear mixed effects model in R.

We investigated the expression of a panel of Sertoli, granulosa and germ cell genes shown previously to be affected by changes in DMRT1 expression in rodent studies. Second-trimester testis tissue exposed to miR536, miR641 or scrambled control virus and cultured as above was assessed. For Sertoli cell associated genes, a significant reduction in *SOX8* expression was demonstrated following exposure to either miR536 (34%, *P* = 0.031) or miR641 (36%, *P* = 0.026), whilst expression of *SOX9* was unaffected (Fig. 3A). A significant increase in *AMH* expression (100%, *P* = 0.033) in the miR536-exposed tissue and downregulation of *CYP26B1* (38%, *P* = 0.05) and *PTGDS* (30%, *P* = 0.0076) in the miR641-exposed samples was also observed. Investigation of granulosa cell associated genes revealed a significant downregulation in *R-spondin 1* expression in tissue transduced with either miR536 (76% reduction, *P* < 0.0001) or miR641 (49% reduction, *P* = 0.046) DMRT1-targeted viruses. In contrast, upregulation of *ESR1* (283%, *P* = 0.019) was seen in response to miR536 transduction (Fig. 3B). There were no changes in expression of the granulosa cell marker, *FOXL2*. Analysis of germ cell associated genes demonstrated a significant increase in the expression of the pluripotency gene *OCT4* (233%, *P* < 0.001) in the miR536-exposed tissue compared to the scrambled control (Fig. 3C).

### DMRT1 repression results in ‘focal dysgenesis’ in second-trimester human fetal testes

**Figure 4:**
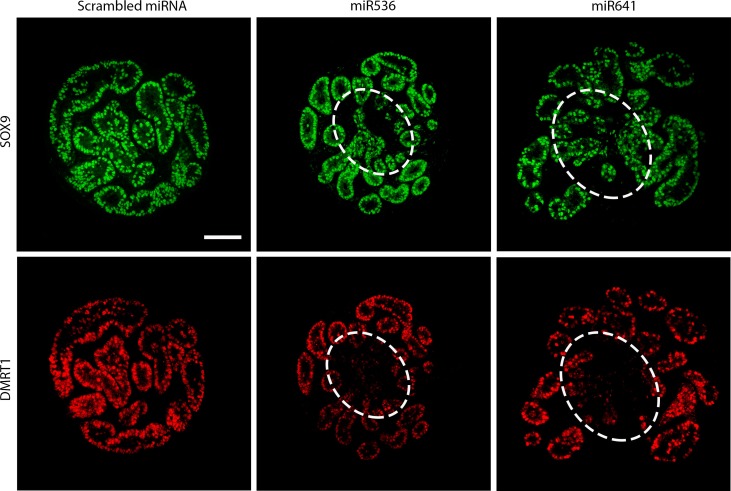
DMRT1 knockdown in vitro results in disruption of seminiferous cords in second-trimester human fetal testis tisse. Immunofluorescence analysis of the expression of SOX9 (Sertoli cells; green) and DMRT1 (Sertoli and germ cells; red) in fetal second-trimester testis tissue cultured for 13 days following an initial 24 h transduction with either scrambled miRNA, miR536 or miR641 lentiviral constructs. Regions of disruption of seminiferous cords can be identified (broken white line) where DMRT1 expression has been reduced but SOX9 expression has been retained. Scale bar: 100 μM.

We further investigated the morphology of first and second-trimester human fetal testis tissues in which we had identified loss of DMRT1 in Sertoli cells (DMRT1^−^/SOX9^+^) after miRNA repression. We used SOX9 to identify Sertoli cells, based on our earlier finding of unchanged mRNA expression (Fig. 3). Morphology of first trimester tissues was similar to the corresponding (scrambled) controls (Fig. 1A). However, in second-trimester human fetal testes, we observed areas of disrupted seminiferous cord structure in regions where DMRT1 expression had been lost in Sertoli cells (Fig. 4).

To determine the longer-term effects of DMRT1 repression on these areas containing disrupted seminiferous cords, a xenograft approach was used. Second-trimester human fetal testis tissue was transduced as described for the *in vitro* experiments using miR536, miR641 or scrambled control. After 24 h of viral exposure in culture, the tissue pieces were transplanted into castrate CD1-nude mice hosts and retrieved 4–6 weeks later. Viral uptake and stable transduction was confirmed by immunofluorescent analysis of mCherry expression in the retrieved grafts (Supplementary Fig. S2). The lack of mCherry staining in the peripheral mouse tissue confirmed viral transduction was restricted to the grafted human fetal tissue and provided an internal control for the experimental analysis.

The overall morphological appearance of the majority of the xenografted tissues was similar to that of the pre-graft control from the same fetus, and seminiferous cord structure was largely maintained in tissues exposed to the scrambled miRNA control, or to miR536 or miR641 (Supplementary Fig. S3A). Cell proliferation (Ki67^+^) was similar between treatment groups, and apoptotic cells (Cleaved Caspase 3^+^; CC3) were rarely identified, indicating that all of the xenografted tissues remained healthy (Supplementary Fig. S3B).

**Figure 5:**
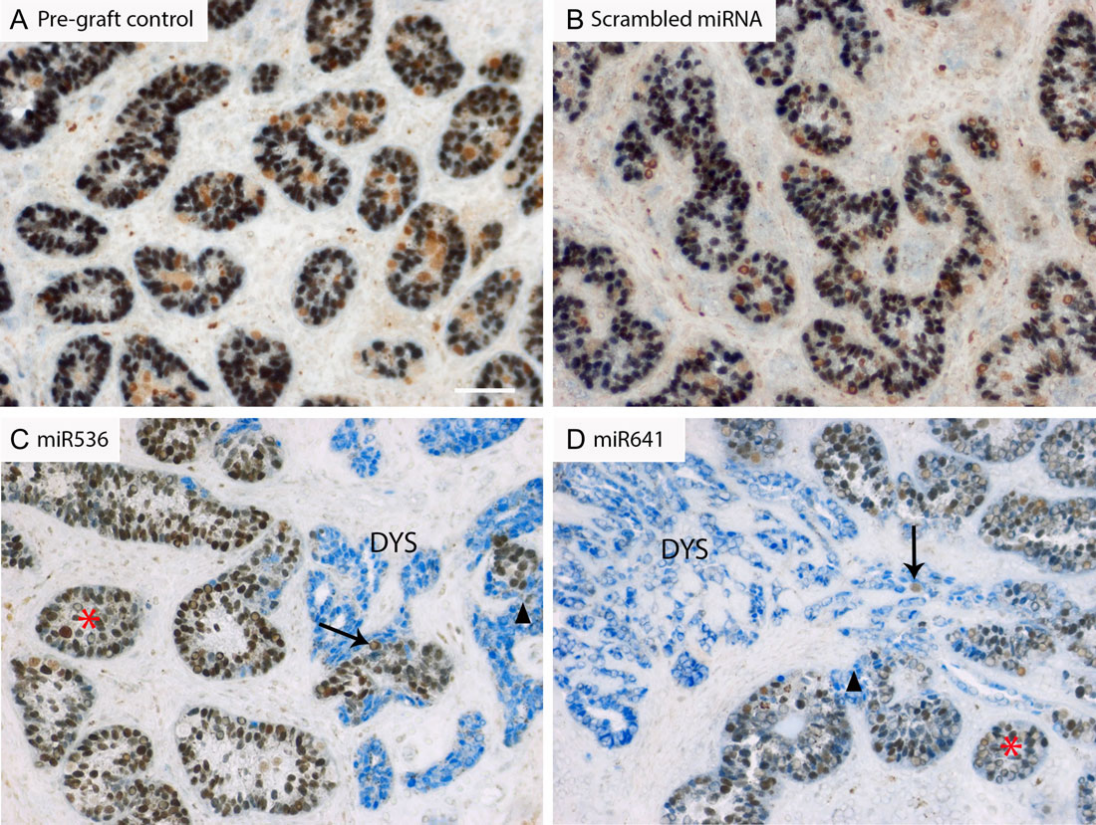
DMRT1 knockdown results in ‘focal testicular dysgenesis’ in second-trimester human fetal testis xenografts. Double immunohistochemistry for DMRT1 (brown; Sertoli and germ cells) and SOX9 (blue; Sertoli cells) in (**A**) pre-graft control, or after xenografting for 6 weeks following an initial 24 h transduction period in culture with (**B**) scrambled miRNA, (**C**) miR536 or (**D**) miR641. Red asterisk = normal seminiferous cord structure, black arrowheads show areas of cord breakdown and the absence of DMRT1 protein; black arrows indicate germ cells and DYS identifies focal dysgenetic areas in which there has been a complete breakdown of seminiferous cords. Scale bar: 50 μM.

We performed immunohistochemical co-localization to identify DMRT1 protein expression in Sertoli (SOX9^+^) cells of xenografted human fetal testis tissues. Co-localization of DMRT1 and SOX9 was observed in all Sertoli cells in all of the control tissues, including pre-graft and scrambled control tissues (Fig. 5A,B), whilst DMRT1^+^/SOX9^−^ cells (presumed to be germ cells) were also evident. Exposure to either miR536 or miR641 resulted in areas containing SOX9^+^/DMRT1^−^ cells in which there was a loss of normal seminiferous cord structure, i.e. focal dysgenesis (Fig. 5C,D); such areas were not observed in either untreated or scrambled control grafts from the same fetuses. In total, sections from 4/6 of the miR536 or miR641-exposed second-trimester fetuses exhibited areas of focal dysgenesis, which was apparent in multiple grafts from individual fetuses (Supplementary Fig. S4).

**Figure 6:**
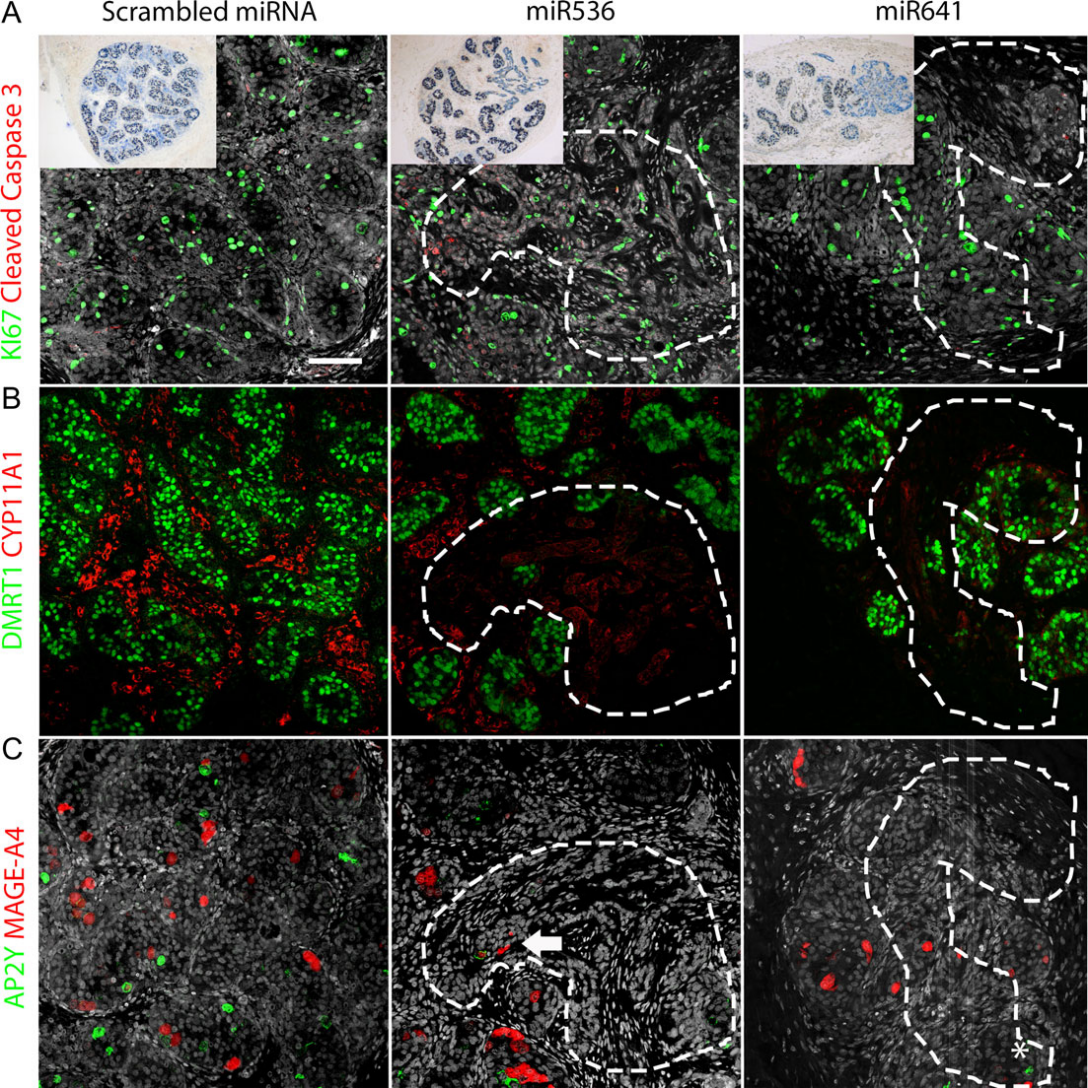
Germ and somatic cell composition and function is altered in ‘focal dysgenetic’ regions of second-trimester human fetal testis resulting from DMRT1 knockdown. Double immunofluorescence for (**A**) Ki67 (green; proliferation marker) and Cleaved Caspase 3 (red; apoptotic marker), (**B**) DMRT1 (green; Sertoli and germ cells) and CYP11A1 (red; Leydig cells), and (**C**) AP2γ (green; gonocytes) and MAGE-A4 (red; pre-spermatogonia). Nuclear counterstain (gray; DAPI/Hoescht). Focal dysgenetic areas are indicated by dashed white lines. White asterisk highlights a single AP2γ+ germ cell within a dysgenetic area (bottom right panel), White arrow indicates a surviving MAGE-A4+ germ cell within a dysgenetic area. Scale bar: 50 μM.

To analyze the focal dysgenetic areas further, we investigated cellular proliferation, apoptosis, and germ and somatic cell marker expression and compared them to control tissues (Fig. 6). Cellular proliferation (Ki67^+^) was similar in dysgenetic areas of miR536/miR641-exposed testes compared either to non-dysgenetic areas or to scrambled control tissues, whilst apoptotic (CC3^+^) cells were infrequently identified across all exposure groups (Fig. 6A and Supplementary Fig. S3B). Leydig cell expression of the steroidogenic enzyme CYP11A1 was seen throughout the interstitial space in scrambled control tissue, consistent with non-exposed controls. However, in the sections of miR536/miR641-exposed tissues containing focal dysgenetic areas, CYP11A1 expression appeared to be reduced in regions surrounding the seminiferous cords with a ‘normal’ appearance and was minimal or absent in the regions surrounding focal dysgenetic areas. Furthermore, within the dysgenetic regions (devoid of DMRT1^+^ Sertoli cells), CYP11A1 expression was completely absent (Fig. 6B).

We investigated the expression of germ cell markers, AP2γ (gonocyte) and MAGE-A4 (pre-spermatogonia) in both scrambled controls and testis tissue with focal DMRT1 repression. In scrambled control tissues, we identified frequent AP2γ^+^ and MAGE-A4^+^ germ cells within the seminiferous cords, similar to previous studies in second-trimester human fetal testis tissues (Mitchell *et al.*, 2008). However, in the dysgenetic areas of the miR536/miR641-exposed xenografts, AP2γ^+^ cells were rarely observed (Fig. 6C). MAGE-A4^+^ cells were identified in seminiferous tubules on the periphery of the dysgenetic regions, but were rarely identified within the focal dysgenetic areas (Fig. 6C).

**Figure 7:**
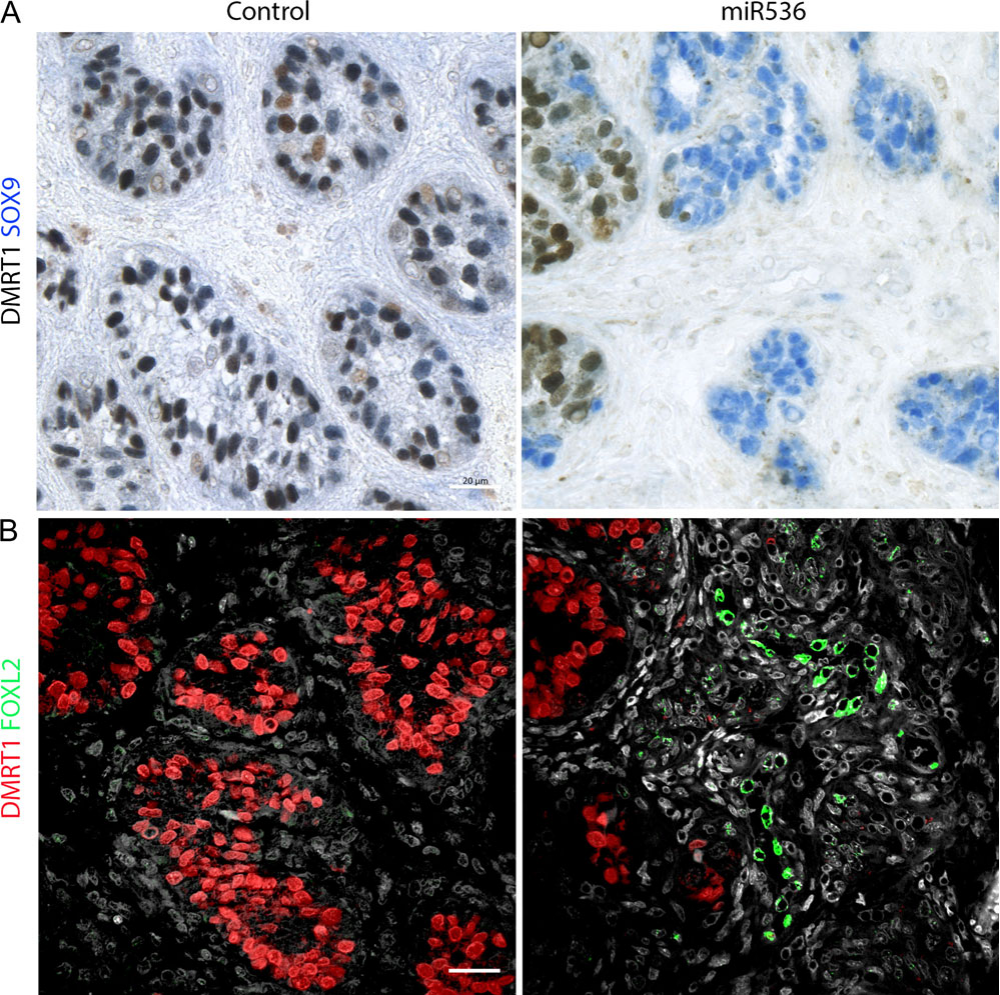
DMRT1 knockdown results in FOXL2 expression in a region of ‘focal dysgenesis’ in a second-trimester human fetal testis xenograft. FOXL2 protein expression in xenografts of control and DMRT1-miRNA transduced second-trimester human fetal testis tissue. (**A**) Double immunohistochemistry for SOX9 (Blue) and DMRT1 (brown) identifies regions of loss of DMRT1 with ‘Focal Dysgenesis’. (**B**) FOXL2 immunoexpression (green) in scattered cells in one fetus in an area devoid of DMRT1 (red) expression following initial transduction with miR536. Scale bar: 20 μM.

Previous studies in rodents have demonstrated that downregulation of DMRT1 expression in the male gonad can result in male to female sex reversal with trans-differentiation of the Sertoli cells (SOX9^+^) to granulosa-like cells (FOXL2^+^) cells (Matson *et al.*, 2011). To investigate the possibility of somatic cell trans-differentiation in the long-term xenografted tissue, we performed immunofluorescent analysis for FOXL2 expression in control and dysgenetic tissue samples. FOXL2 expression was not observed in any of the scrambled control tissues; however, we identified FOXL2 expression in tissue from one of five fetuses from miR536 transduced testes, and this was within a dysgenetic region devoid of DMRT1 expressing cells (Fig. 7).

## Discussion

Impairment of normal development of the human gonad during fetal life may have implications for future reproductive potential and the development of gonadal tumors (Looijenga *et al.*, 2010; Jørgensen *et al.*, 2015a; Hersmus *et al.*, 2017); this has been the foundation for the TDS hypothesis (Skakkebaek *et al.*, 2016). An increasing number of genetic mutations associated with DSD are being described in humans; however, much of our understanding of these mutations and the mechanism(s) by which they result in testicular dysfunction is based on rodent studies. Mutations/deletions in the short arm of Chromosome 9, where *DMRT1* is located is strongly associated with DSD and sex-reversal in humans (Raymond *et al.*, 1999b; Ottolenghi *et al.*, 2000; Ounap *et al.*, 2004; Vinci *et al.*, 2007) and a recent study has reported a patient with XY sex-reversal associated with a point mutation in DMRT1 (Murphy *et al.*, 2015). Recent evidence from rodent studies show that inducing *DMRT1* mutations, even in postnatal life, can lead to trans-differentiation of the gonad from a testicular to an ovarian phenotype, demonstrating plasticity in gonadal fate throughout life (Matson *et al.*, 2011). In this study, we developed a novel system of lentiviral mediated miRNA manipulation to target a gene of interest in the human fetal testis, which combined *in vitro* culture with a xenografting approach to investigate the short- and long-term consequences of manipulation. This approach was used to determine the effects of reduced DMRT1 expression on testicular development and assess whether plasticity of gonadal fate exists in humans as in rodents. We show that DMRT1 expression can be repressed in Sertoli cells of the human fetal testis resulting in altered expression of gonad-specific genes and proteins, indicating a move away from a fully differentiated male phenotype, and potentially towards a more ovarian-like phenotype. Moreover, we demonstrate that DMRT1 repression in the human fetal testis results in disruption of prior-formed seminiferous cords with development of focal testicular dysgenesis. A similar disruption of prior-formed seminiferous cords has been reported also in rats after exposure to an environmental chemical (dibutyl phthalate), and shown to result in focal dysgenesis (Lara *et al.*, 2017).

We have validated a dynamic model system for genetic manipulation of the human fetal testis by transducing first and second-trimester testis tissue with miRNAs targeting the DMRT1 gene at two separate regions (miR536 and miR641). This resulted in repression of DMRT1 expression in focal regions of exposed human fetal testis tissue pieces after 14 days of culture. DMRT1 repression was observed at the protein level in both first and second-trimester human fetal testes with either DMRT1-targeted miRNA, although the *DMRT1* mRNA transcript level was only reduced in second-trimester tissue transduced with the miR641 transcript. The lack of identifiable transcript alteration in both first and second-trimester tissues with each of the miRNAs is not unusual given that miRNA can result in either translational repression or endonucleolytic activity resulting in mRNA degradation and is dependent on the level of complementarity with the target mRNA (Schmitter *et al.*, 2006). The targeting region of the RT-qPCR primers used to detect repression in relation to the miRNA target site can also influence the ability to identify gene repression at the mRNA level (Holmes *et al.*, 2010). Although we have previously shown that it is possible to inhibit and promote signaling pathways in fetal testis gonad culture using chemical manipulation (Jørgensen *et al.*, 2015b), to the best of our knowledge there are no publications showing successful ‘long-term’ genetic manipulation of the human fetal gonad. We have shown that repression of DMRT1 activity persists over several weeks in this study by using xenografting techniques to extend the experimental time from 14 days (in culture) to 4–6 weeks (via xenografts), after which time SOX9^+^/DMRT1^−^ cells were still frequently identified in testis tissue exposed to DMRT1-targeted miRNA. The location of the DMRT1 repression appears to relate to the degree of viral penetration within the tissue and the resulting focal areas of dysgenesis are likely to result from proliferation of the DMRT1^−^ Sertoli (SOX9^+^) cells. The focal distribution of DMRT1 repression and corresponding dysgenesis also provides a useful internal control for identifying specific effects of gene repression within each tissue sample.

It has previously been shown that DMRT1 is important for maintaining the testicular phenotype by preventing Sertoli-to-granulosa cell trans-differentiation in the postnatal mouse testis by inhibiting the expression of key ‘female pathway’ genes whilst promoting the continued expression of ‘male pathway’ promoting genes (Matson *et al.*, 2011; Minkina *et al.*, 2014). To examine potential effects of DMRT1 repression in Sertoli cells on genes known to be linked to maintenance of gonad sex identity, we used RT-qPCR to investigate expression of Sertoli, granulosa and germ cell specific genes. Notably, *SOX8* expression was reduced in both the miR536 and miR641-exposed tissues. This is in agreement with a rodent study which elegantly demonstrated that when DMRT1 was knocked out in both prepubertal and adult male mice, it resulted in down- regulation of *SOX8* and *SOX9* (Matson *et al.*, 2011). Unlike Matson *et al.* (2011) we did not observe a reduction in *SOX9* expression following DMRT1 knockdown in postnatal mice, although this is in keeping with studies involving *Dmrt1* deletion in mice, in which S*ox9* expression is initially independent of DMRT1 (Matson *et al.*, 2011). It is possible that loss of *Sox9* after *Dmrt1* ablation is a secondary consequence of the upregulation of ovarian genes(s), such as *Foxl2*, which does not occur until postnatal life (Matson *et al.*, 2011). Whilst SOX9 is essential for sex determination, it has been shown in some studies to be dispensable for the maintenance of sexual phenotype beyond fetal life (Chaboissier *et al.*, 2004), although this conclusion does not take into consideration the importance of continued expression of DMRT1 or SOX8 (Barrionuevo *et al.*, 2016). It is possible that DMRT1 and SOX9 provide a protection system for the maintenance of sexual phenotype in the male gonad during different stages of development. In contrast, SOX8, remains a potential candidate as a key sexual maintenance gene in the male gonad (Barrionuevo *et al.*, 2009, 2016). Furthermore, mutations in SOX8 have recently been demonstrated in humans with DSD and a lack of testis determination (Portnoi *et al.*, 2018).

Other than *SOX8*, no other ‘male pathway’ genes were found to be differentially altered after knockdown of DMRT1, although *CYP26B1* and *PTGDS* (Prostaglandin D2 synthase) were both down-regulated in the miR641 transduced samples. *CYP26B1* and *DMRT1* are both involved in preventing premature initiation of meiosis in rodent fetal testes by repression of retinoic acid (RA) signaling, including inhibiting the expression of the pre-meiosis marker STRA8 (Bowles *et al.*, 2006; Koubova *et al.*, 2006; Matson *et al.*, 2010). In addition, the *CYP26B1* expression which is activated by SOX9 in the fetal mouse testis was recently shown to also antagonize the ovarian differentiation pathway possibly through the involvement of DAX1 (Bowles *et al.*, 2018). In terms of genes associated with an ovarian phenotype, the granulosa cell marker *R-spondin *1 was down-regulated despite repression of DMRT1, whilst there was no change in *FOXL2* expression at a transcript level. However, we did identify FOXL2 protein expression in tissue from one of the xenografted fetuses in an area of the tissue in which DMRT1 was repressed, whilst no expression of FOXL2 was detected in the control xenografts from the same fetus. This might indicate that a longer time-period of DMRT1 repression is required for FOXL2 expression to be initiated, as analysis of xenografts occurred 4–6 weeks later than for the cultured tissues. The rare finding of FOXL2 expression in the xenografted tissue could indicate that there are several pathways or factors working in concert to maintain a gonadal phenotype, a concept that is supported by descriptions of significant inter-individual variation in the occurrence of male to female sex reversal in patients with various forms of XY gonadal dysgenesis (Bagheri-Fam *et al.*, 2015). In addition, the potential for DMRT1 repression to induce FOXL2 expression may be reduced in the xenografts due to the fact that DMRT1 is only lost in a proportion of the Sertoli cells or that focal expression of these FOXL2^+^ cells may be restricted by a low rate of their proliferation.

A key finding of the study was the demonstration of regions of focal dysgenesis in the second-trimester human fetal testis as a result of DMRT1 repression. Whilst seminiferous cord formation progressed in first trimester human fetal testes in which DMRT1 was repressed, DMRT1 repression in second-trimester tissue, in which the seminiferous cords had already formed, resulted in localized disruption of cord structure after short-term *in vitro* culture. Long-term maintenance of the testis tissue in xenografts showed further progression of cord breakdown into regions of focal dysgenesis. These findings indicate a role for DMRT1 in the maintenance of testicular phenotype following sex determination and seminiferous cord formation in the human testis. This is in keeping with data from rodent studies where *Dmrt1* null XY mice initially develop testes but subsequently undergo male to female sex reversal (Raymond *et al.*, 2000). Whilst the present study suggests that DMRT1 is required to maintain the testicular phenotype during fetal life in humans, studies in *Dmrt1*^*fl/fl*^ mutant mice show that trans-differentiation towards a female phenotype does not begin until around postnatal Day 8 (Minkina *et al.*, 2014). Furthermore, the latter authors demonstrated that trans-differentiation was more pronounced in the *Dmrt1*^*−*^/*Sox9*^*-*^ double knockout, evidenced by increased numbers of FOXL2^+^ cells by 14 days and almost complete sex reversal at 5 weeks compared to the *Dmrt1*^*-*^ only mutant. In the present study SOX9 expression was unaffected and therefore it is unclear whether simultaneous loss of SOX9 would lead to a more frequent or severe gonadal phenotype. Taken together, these results suggest that, similar to the mouse, DMRT1 is not essential for seminiferous cord formation in the human fetal testis, but is required for subsequent maintenance of the seminiferous cords and for the prevention of trans-differentiation to an ovarian phenotype. In the context of testicular dysgenesis syndrome (TDS) disorders, the present results suggest that the focal dysgenesis which is frequently observed in the testes of patients with reproductive disorders (Skakkebaek *et al.*, 2016), could arise as the result of the breakdown of already formed seminiferous cords, as recently documented in an animal model of TDS (Lara *et al.*, 2017; van den Driesche *et al.*, 2017). Moreover, the fact that DMRT1-miRNA-induced focal dysgenesis was associated with evidence for impaired Leydig cell steroidogenesis, as indicated by reduced CYP11a1 expression, fits with the clinical (Skakkebaek *et al.*, 2016) and rodent experimental (van den Driesche *et al.*, 2017) data, and this is considered to be the underlying cause of some of the TDS disorders.

The present findings have important implications for the study of DSD and TDS. Focal testicular dysgenesis is frequently found in patients with these disorders and there is a strong association between DSD (Y-chromosome^+^) and testicular germ cell tumors (TGCC; Looijenga *et al.*, 2010; Jørgensen *et al.*, 2015a; Hersmus *et al.*, 2017). Indeed, in humans, the presence of focal dysgenesis has been shown to correlate with the presence of germ cell neoplasia in situ (GCNIS), the precursor to TGCC (Hoei-Hansen *et al.*, 2003; van den Driesche *et al.*, 2017) and genetic variations in or near to DMRT1 have also been associated with TGCC in GWAS studies (Turnbull *et al.*, 2010; Kanetsky *et al.*, 2011). Indeed, DMRT1 is aberrantly expressed in the testicular cancer precursor germ cell neoplasia in situ (GCNIS) as part of a general dysregulation of the mitosis-meiosis switch in these cells (Jorgensen *et al.*, 2013; Sharpe and Mitchell, 2013). Further support for a potential role of DMRT1 in the pathogenesis of TGCC is the present finding that *PTGDS* gene expression was also down-regulated following DMRT1 repression (miR641) in the human fetal testis. PTGDS expression has been associated with inhibition of cellular proliferation and migration in the human testicular germ cell cancer line NT2 (Wu *et al.*, 2012a; Shyu *et al.*, 2013). GCNIS cells (and gonocytes) express markers including the pluripotency factors OCT4 and AP2γ: we have shown a significant increase in *OCT4* gene expression following DMRT1-miRNA (miR536) repression. Furthermore, in xenografts, repression of DMRT1 resulted in focal dysgenesis similar to that described in patients with TGCC (Hoei-Hansen *et al.*, 2003). However, germ cells expressing AP2γ were rarely observed in the dysgenetic regions of xenografted human fetal testes in which DMRT1 had been repressed. Whether the few remaining germ cells within these dysgenetic regions undergo pre-neoplastic change requires further investigation.

More widely, the use of this novel system for genetic manipulation in human fetal testis provides a unique opportunity to determine the role of other genes or gene networks that may be involved in testicular development in health and disease (including DSD and TDS). This may include studies to determine the human-relevance of results from rodent models or those that can investigate the pathogenicity and potential mechanism(s) for mutations identified in patients with these disorders.

In conclusion, the present study has utilized a novel approach for genetic manipulation in the human fetal testis, resulting in the first direct evidence that DMRT1 is required for testicular differentiation in humans. We have shown that DMRT1 repression results in alterations in expression of key genes involved in gonadal development, consistent with DMRT1 preventing trans-differentiation of Sertoli cells to a more granulosa-like cell type in the human testis. Furthermore, we have demonstrated that repression of DMRT1 results in focal dysgenesis in the human fetal testis. These findings relating to DMRT1 effects, coupled with the potential use of this system to investigate the effects of manipulation of other genes, may have important implications for understanding the origins of DSD, TGCC and related disorders in humans.

## Supplementary Material

Supplementary Figure 1

Supplementary Figure 2

Supplementary Figure 3

Supplementary Figure 4

Supplementary Table 1

Supplementary Table 2

Supplementary Table 3
