# Supplementary material for: DMRT1 repression using a novel approach to genetic manipulation induces testicular dysgenesis in human fetal gonads
Source: Hum Reprod. 2018 Sep 29;33(11):2107–21. doi: 10.1093/humrep/dey289 (PMC6195803; doi:10.1093/humrep/dey289)
Supplement: Supplementary Figure 1 [file dey289suppl_figure1.pdf]

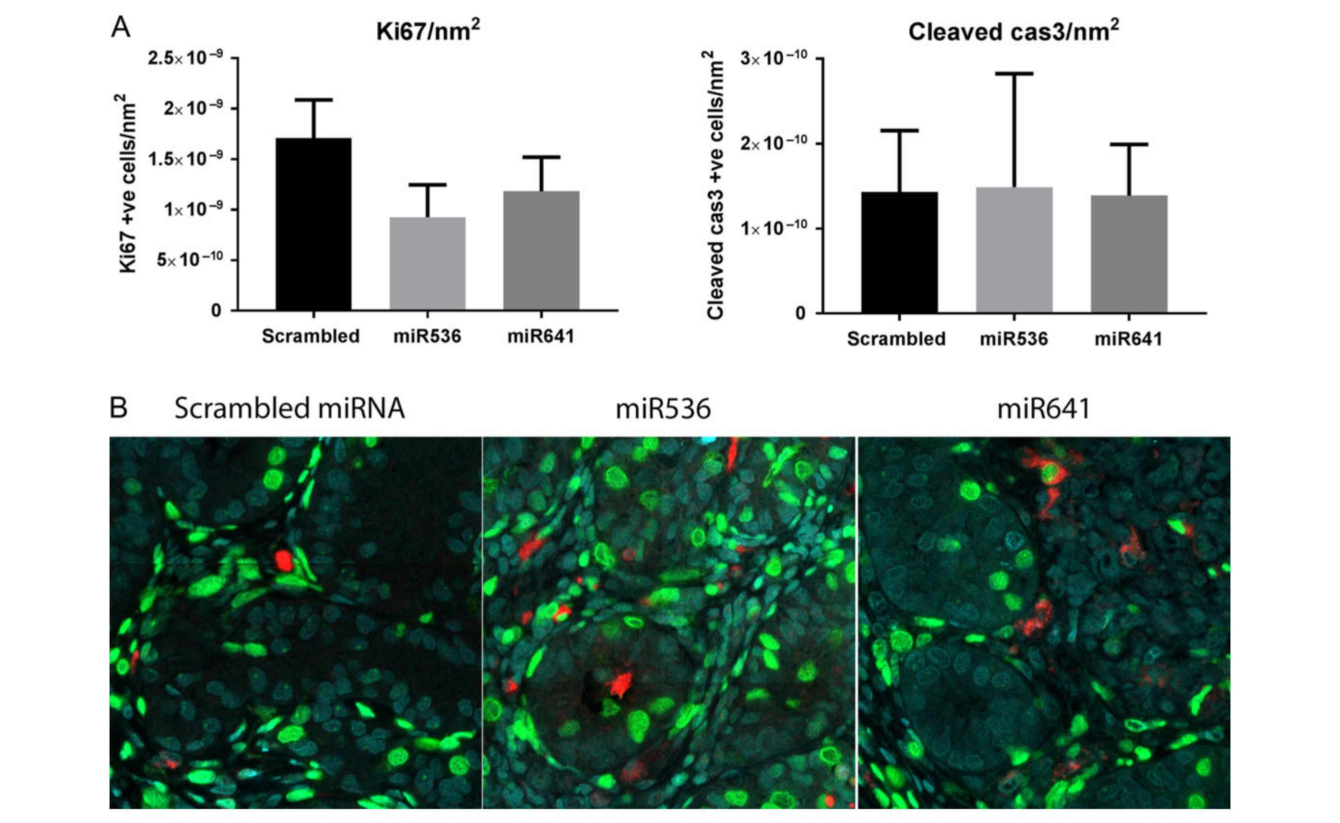

Supplementary Figure S1 roliferation (Ki67+) and apoptosis (Cleaved Caspase 3+) in human fetal testis tissue after lentiviral transduction and 13 day hanging drop culture. (**A**) Quantification of Ki67+ and Cleaved Caspase 3+ in first trimester human fetal testis. (**B**) Immunofluorescence for Ki67 (Green) and Cleaved Caspase 3 (red) in second-trimester human fetal testis tissue. Nuclear counterstain with DAPI.
