# Supplementary material for: DMRT1 repression using a novel approach to genetic manipulation induces testicular dysgenesis in human fetal gonads
Source: Hum Reprod. 2018 Sep 29;33(11):2107–21. doi: 10.1093/humrep/dey289 (PMC6195803; doi:10.1093/humrep/dey289)
Supplement: Supplementary Figure 2 [file dey289suppl_figure2.pdf]

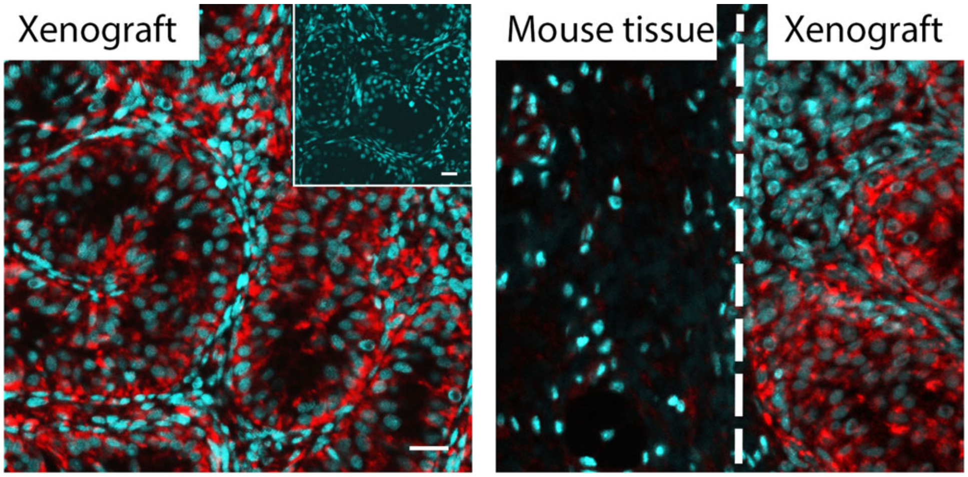

Supplementary Figure S2 Analysis of viral uptake in xenografted human fetal testis tissue. Single immunofluorescent analysis showing mCherry protein expression (Lentiviral Fluorescent Tag: red) in xenografted second-trimester human tissue (left panel) and absent from the mouse connective tissue surrounding the xenograft (right panel). Inset negative control. Nuclear counterstain with DAPI. Scale bar: 20 μM.
