# Supplementary material for: DMRT1 repression using a novel approach to genetic manipulation induces testicular dysgenesis in human fetal gonads
Source: Hum Reprod. 2018 Sep 29;33(11):2107–21. doi: 10.1093/humrep/dey289 (PMC6195803; doi:10.1093/humrep/dey289)
Supplement: Supplementary Figure 3 [file dey289suppl_figure3.pdf]

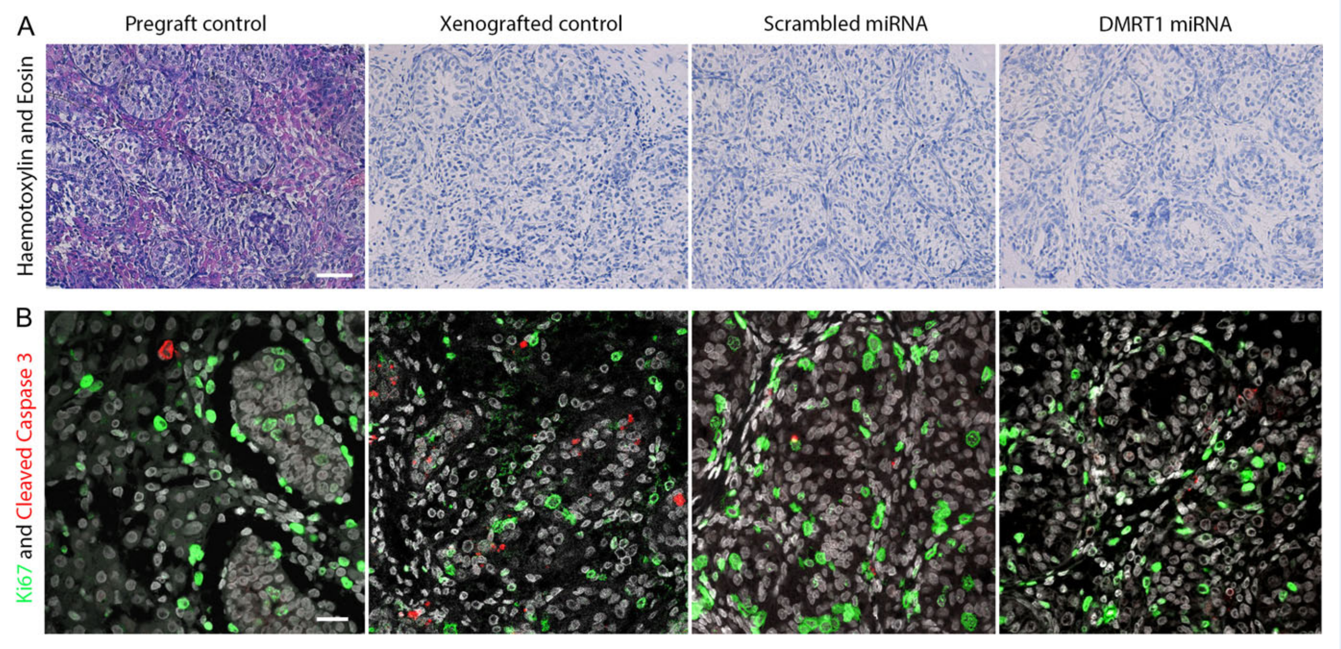

Supplementary Figure S3 (**A**) Histological appearance (H + E staining) of pre-graft and xenografted second-trimester testis tissue transduced with scrambled miRNA or DMRT1-miRNA lentiviral constructs 536 and 641. (**B**) Double immunofluorescence for proliferation (Ki67; green) and apoptosis (cleaved caspase; red). Scale bar A: 20 µM and B: 50 µM.
