# Supplementary material for: DMRT1 repression using a novel approach to genetic manipulation induces testicular dysgenesis in human fetal gonads
Source: Hum Reprod. 2018 Sep 29;33(11):2107–21. doi: 10.1093/humrep/dey289 (PMC6195803; doi:10.1093/humrep/dey289)
Supplement: Supplementary Figure 4 [file dey289suppl_figure4.pdf]

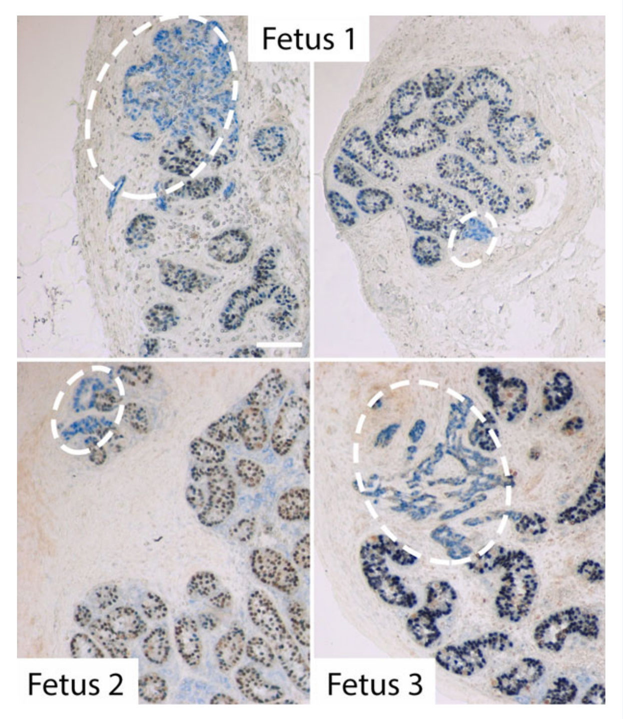

Supplementary Figure S4 Double immunohistochemistry for DMRT1 (Sertoli and germ cells; brown) and SOX9 (Sertoli cells; blue) protein in DMRT1-miRNA transduced tissue. Areas of dysgenesis in sections of xenografted tissue from three further human fetuses are shown (broken white line). Scale bar 100 μM.
