# Supplementary material for: DMRT1 repression using a novel approach to genetic manipulation induces testicular dysgenesis in human fetal gonads
Source: Hum Reprod. 2018 Sep 29;33(11):2107–21. doi: 10.1093/humrep/dey289 (PMC6195803; doi:10.1093/humrep/dey289)
Supplement: Supplementary Table 1 [file dey289suppl_table1.pdf]

Human Reproduction, 2018
doi:10.1093/humrep/dey289

human
reproduction

SUPPLEMENTARY DATA

Supplementary Table SI **miRNA Oligonucleotides for Scrambled and miRs 536 and 641**

Name	Oligonucleotide sequence: mature miR, loop, 1–8, 11–21 21 mer target Scr 5′-
**Scrambled**	5′-GAAATGTACTGCGCGTGGAGACGTTTTGGCCACTGACTGACGTCTCCACGCAGTACATTT-3′
**miR536**	5′-TGCTGTATCCTGGATGACCATGCGTCGTTTTGGCCACTGACTGACGACGCATGCATCCAGGATA-3′
**miR641**	5′-TGCTGTGTAGTAAGGAAACAGAGACGGTTTTGGCCACTGACTGACCGTCTCTGTCCTTACTACA-3′
