# Supplementary material for: DMRT1 repression using a novel approach to genetic manipulation induces testicular dysgenesis in human fetal gonads
Source: Hum Reprod. 2018 Sep 29;33(11):2107–21. doi: 10.1093/humrep/dey289 (PMC6195803; doi:10.1093/humrep/dey289)
Supplement: Supplementary Table 2 [file dey289suppl_table2.pdf]

Supplementary Table SII **Antibodies used for immunohistochemistry (IHC) or immunofluorescence (IF)**

Primary antibodies	Dilution	Supplier
DMRT1	1:250 (IHC); 1:1000 (IF)	Sigma-Aldrich (HPA027850)
SOX9	1:2000 (IHC); 1:10000 (IF)	Chemicon (AB5535)
FOXL2	1:2000 (IF)	ABCAM Ltd (AB5096)
Ki67	1:100 (IF)	DAKO M7240
Cleaved Caspase 3	1:50 (IF)	Chemicon (AB3623)
Living colors DsRed	1:1000 (IF)	Clontech (632496)
**Secondary Antibodies**		
Chicken anti-rabbit peroxidase	1:200	Santa Cruz Biotechnology (sc-2995)
Chicken anti-mouse peroxidase	1:200	Santa Cruz Biotechnology (sc-2962)
Goat anti-rabbit peroxidase	1:200	Vecto Laboratories (PI-1000)
ImmPRESS™ HRP anti-rabbit IgG (peroxidase)	1 drop/slide	Vector Laboratories (MP-7401)
ImmPRESS™ AP anti-rabbit IgG (Alkaline phosphatase)	1 drop/slide	Vector Laboratories (MP-5401)
