# Supplementary material for: DMRT1 repression using a novel approach to genetic manipulation induces testicular dysgenesis in human fetal gonads
Source: Hum Reprod. 2018 Sep 29;33(11):2107–21. doi: 10.1093/humrep/dey289 (PMC6195803; doi:10.1093/humrep/dey289)
Supplement: Supplementary Table 3 [file dey289suppl_table3.pdf]

Supplementary Table SIII **RT-qPCR primers**

Gene	Forward primer	Reverse primer
VASA	TCTCTGTTCCCGATCACCAT	CGTTGAAATTCTGCGAAACA
SOX9	GTAATCCGGGTGGTCCTTCT	GTACCCGCACTTGCACAAC
dHH	ACATGTTCATCACGGCAATG	AACCCCGACATCATCTTCAA
FGF9	TTCCAGTGTCCACGTGCTTA	GAAGGGGGAGCTGTATGGAT
AMH	GCTGGACACCGTGCCCTTCC	CCTCGAGTTCCGCGGATGGC
RSP29	CGCTCTTGTCGTGTCTGTTCA	CCTTCGCGTACTGACGGAAA
FOXL2	TACTCGTACGTGGCGCTCAT	CTCGTTGAGGCTGAGGTTGT
CYP19A1	GCCGAATCGAGAGCTGTAAT	GAGAATTCATGCGAGTCTGGA
CYP26B1	AGGTAACTCTCCAGGGCCTC	ACACGGTGTCCAATTCCATT
ESR1	CAGGATCTCTAGCCAGGCAC	ATGATCAACTGGGCGAAGAG
ESR2	ACCAAAGCATCGGTCACG	CATGATCCTGCTCAATTCCA
R-spondin	AGAGCAGAGCTCACAGCCTT	CCTGCTGACGTGACAAAAAG
WNT4	AGTTTCTCGCACGTCTCCTC	CTCGTCTTCGCCGTCTTCT
SOX8	GTGGTCCTTCTTGTGCTGC	AGCTCAGCAAGACGCTGG
CTNNB1	ATTGTCCACGCTGGATTTTC	TCGAGGACGGTCGGACT
SOX10	CTTTCTTGTGCTGCATACGG	AGCTCAGCAAGACGCTGG
PTGDS	ACTTGCACATGGACAACGC	CCAACTTCCAGCAGGACAA
SYCP3	TTCATTTTGTGCACCAGTAAGTAGA	TCAGAGCCAGAGATTGAAAACA
OCT4	TACTCCTCGGTCCCTTTCC	CAAAAACCCTGGCACAAACT
DMRT1 536	AAGAAGTGCAACCTGATCGC	CAGGTCAGGTGTGTTCTCCA
DMRT1 641	TGGAGAACAACACCTGACCTG	GATTCCCACCTCCCCAGAA
